# A large‐scale field experiment of artificially caused landslides with replications revealed the response of the ground‐dwelling beetle community to landslides

**DOI:** 10.1002/ece3.9939

**Published:** 2023-03-24

**Authors:** Jumpei Furusawa, Kobayashi Makoto, Shunsuke Utsumi

**Affiliations:** ^1^ Graduate School of Environmental Science Hokkaido University Sapporo Japan; ^2^ Nayoro Research Office, Field Science Center for Northern Biosphere Hokkaido University Nayoro Japan; ^3^ Field Science Center for Northern Biosphere Hokkaido University Sapporo Japan

**Keywords:** cool‐temperate climate, dispersal, ecological drift, ecological restoration, landslide, metacommunity, mixed forest, pitfall trap

## Abstract

Precipitation‐induced landslides, which are predicted to increase under the changing climate, may have large impacts on insect community properties. However, understanding of how insect community properties shift following landslides remains limited because replicated research involving landslides, which are large‐scale disturbances with stochastic natural causes, is difficult. To tackle this issue, we conducted a large‐scale field experiment by artificially causing landslides at multiple sites. We established 12 landslide sites, each 35 m × 35 m, and 6 undisturbed sites in both planted and natural forests and collected ground‐dwelling beetles 1 year later. We found that forest type (i.e., pre‐disturbance vegetation) did not affect the structure of a ground‐dwelling beetle community disturbed by a landslide (landslide community), but the structure of an undisturbed community was affected by forest type. Moreover, the structures of landslide and undisturbed communities were completely different, possibly because landslides create harsh environments that act as an ecological filter. Thus, a niche‐selection process may have a critical role in community assembly at landslide sites. There were no significant differences in species diversity between undisturbed and landslide communities, suggesting that landslides to not reduce species richness overall. However, among‐site variability in species composition was much greater at landslide sites than at undisturbed sites. This result suggests that stochastic colonization predominated at the landslide sites more than undisturbed sites. *Synthesis and applications*. Overall, our results suggest that both deterministic and stochastic processes are critical in community assembly, at least in the early post‐landslide stage. Our large‐scale manipulative field experiment with replications has thus resulted in new insights into biological community properties after a landslide.

## Graphical abstract

To our knowledge, this is the first time that a large‐scale field experiment by artificially causing landslides at multiple sites to examine initial insect diversity responses before plant vegetation recover. We found that among‐site variability was significantly large in response to the landslide treatment and that both deterministic and stochastic processes are critical in community assembly, at least in the early post‐landslide stage. Our large‐scale manipulative field experiment with multiple replicates has thus resulted in new insights into biological community properties after a landslide.

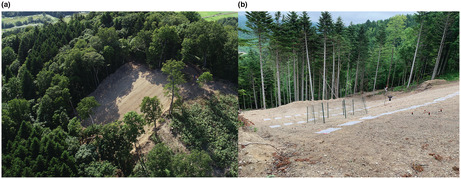

## 1 INTRODUCTION

Large‐scale disturbances caused by global climate change and anthropogenic environmental modification have profound impacts on biodiversity and the provision of vital ecosystem services around the world (Aavik et al., 2021; Barnosky et al., 2012; Seidl et al., 2017). In recent years, the frequency and severity of large‐scale disturbances have increased globally (Patacca et al., 2023; Seidl et al., 2017; Westerling, 2016). While natural disturbances have historically impacted biomes and can contribute to the maintenance of biodiversity (Schowalter, 2012), recent changes in disturbance regimes may result in unprecedented biodiversity loss (Bowd et al., 2023; Johnstone et al., 2016). However, our understanding of the general principles governing the responses of ecological communities to the recent large‐scale disturbances remains incomplete. This knowledge could lead to the development of appropriate conservation and ecosystem management practices as an urgent issue for the maintenance of biodiversity, ecosystem functions, and a sustainable society.

One type of large‐scale disturbance that may increase in frequency because of global climate change is a landslide, which can be triggered by extreme precipitation (Gariano & Guzzetti, 2016; Sidle & Bogaard, 2016). Landslides greatly reduce the quality of biological habitats through changes in both topography and environmental factors, including the loss of huge amounts of surface soil and vegetation (Highland & Bobrowsky, 2008). On the other hand, at local scale, landslides may create habitat heterogeneity, thereby contributing to the maintenance of biodiversity (Geertsema & Pojar, 2007; Remelli et al., 2019). Understanding of how ecological community properties such as species composition and species diversity shift following landslides is still limited for two main reasons.

First, most previous studies of the impacts of landslides have focused primarily on plant communities (Guariguata, 1990; Walker et al., 1996). For example, the process of vegetation recovery after landslides has been monitored (Chen et al., 2020; Yang et al., 2018), and how environmental factors such as soil conditions affect the post‐landslide vegetation recovery rate has been examined (Lin et al., 2005; Shiels et al., 2008). A very few studies have examined non‐plant communities such as arthropods (Hao‐Chiang et al., 2017), which may rapidly colonize and alter biophysical conditions of landslide‐degraded habitats in advance of plant colonization. For example, ground‐dwelling beetles can influence soil quality though relocating waste by vertebrates and transporting fungi (Nichols et al., 2008; Vašutová et al., 2019). In this regard, it should be noted that arthropods, the most diverse eukaryotic group on Earth, have a critical role in ecosystem functioning (Prather et al., 2013). Second, because a landslide is a large‐scale disturbance caused by a stochastic natural event such as an earthquake or heavy rainfall (Sidle & Bogaard, 2016), studies of the impacts of landslides suffer a methodological limitation. Each study has typically been conducted in a single disturbed locality, each with different geographic and environmental conditions (e.g., climate and vegetation), without replications or experimental treatments (Schowalter, 2012). As a result, general patterns in how community properties shift in response to landslides and in how environmental factors influence post‐landslide community assemblages are poorly understood. To address these problems, large‐scale field experiments with replications of artificially caused landslides are needed that examine a wide range of ecological communities, in addition to vegetation communities.

Community assembly occurs by both stochastic (i.e., ecological drift) and deterministic (i.e., niche selection) processes (Chase & Leibold, 2003; Chave, 2004; Chesson, 2000; Hubbell, 2001). Although stochastic and deterministic processes are not mutually exclusive, their relative importance varies over time and space under different environmental conditions (Chase, 2010; Chave, 2004; Leibold & McPeek, 2006). Chase (2007) showed that, in harsh environments, niche selection predominantly filters out species rather than the stochastic process of ecological drift. Thus, the environmental harshness that follows landslides can be expected to result in niche‐assembled communities. Although pre‐disturbance vegetation may affect post‐disturbance community assemblages because of remaining propagules (Bergeron et al., 2017; Johnstone et al., 2016), this effect may be weaker following a landslide than after other types of disturbances such as clear‐cutting and forest fires because both natural and artificial landslides often completely remove the surface soil along with any propagules and insect eggs or larvae.

In this study, we examined the community assembly response to replicated experimental landslides. We conducted a large‐scale field experiment with replications of artificially caused landslides and investigated communities of ground‐dwelling beetles, which may rapidly colonize a disturbed area regardless of whether all vegetation is removed. Ground‐dwelling beetles are useful bioindicators because of their quick response to environment change (Rainio & Niemelä, 2003). Further, some of ground‐dwelling beetles are known as seed predators and can mediate seed dispersal (Ali et al., 2022; de Vega et al., 2011; Griffiths et al., 2016; Müller et al., 2022), and even some fungi are dispersed by ground‐dwelling beetles (Heitmann et al., 2021; Vašutová et al., 2019). Moreover, plant growth enhancement by some species of ground‐dwelling beetles through improving soil quality was reported (Nichols et al., 2008). Thus, because the initial short‐term responses of ground‐dwelling beetles to disturbances may affect the subsequent succession of plants and other taxa, general insights into their initial responses gained by our experimental approach can provide a basis for ecological restoration. In addition, our experimental approach allows us to test the effects of the assembly process (i.e., niche selection or ecological drift) and the effects of pre‐disturbance vegetation (i.e., natural or planted forest) following a disturbance realistically mimicking a landslide disturbance in natural ecosystems. Specifically, we address the following questions: (1) How does forest type affect the beetle community structure under undisturbed conditions and post‐landslide conditions? (2) Do landslide environments act as a filter, resulting in a different community structure between landslide and undisturbed conditions? (3) Does the occurrence of a landslide reduce species diversity and among‐site variability of communities of ground‐dwelling beetles?

## 2 MATERIALS AND METHODS

### 2.1 Landslide experiment

This study was conducted in the Teshio, Nakagawa, and Uryu Experimental Forests of Hokkaido University in northern Hokkaido, Japan. The dominant tree species in these cool‐temperate zone experimental forests are birch (*Betula ermanii*, and *Betula platyphylla* var. *japonica*), Japanese oak (*Quercus mongolica* var. *grosseserrata*), painted maple (*Acer mono*), and Sakhalin fir (*Abies sachalinensis*). In natural forests, the understory is dominated by dwarf bamboo (*Sasa senanensis*). In each experimental forest (hereafter referred to as “locality”), we established four landslide treatment sites, consisting of three natural forest sites and one site in a planted forest of *A*. *sachalinensis* (Table 1). We also established two undisturbed sites as controls in each experimental forest; one was in a natural forest and the other was in a planted forest of *A*. *sachalinensis* (Table 1). Thus, a total of 12 landslide treatment sites and 6 undisturbed sites were established in the three localities.

**TABLE 1:** Description of study sites.

Locality	Forest‐type	Treatment	Latitude (°N)	Longitude (°E)	Slope (%)	Aspect (°)
Teshio	Planted	Undisturbed	44.930	141.976	N/A	233.511
Teshio	Natural	Undisturbed	44.983	141.993	N/A	221.700
Teshio	Planted	Landslide	44.931	141.977	21	256.706
Teshio	Natural	Landslide	44.986	141.994	24	134.490
Teshio	Natural	Landslide	44.986	141.995	23	133.390
Teshio	Natural	Landslide	44.987	141.996	21	129.815
Nakagawa	Planted	Undisturbed	44.786	142.254	N/A	244.146
Nakagawa	Natural	Undisturbed	44.792	142.249	N/A	231.540
Nakagawa	Planted	Landslide	44.785	142.255	26	87.259
Nakagawa	Natural	Landslide	44.786	142.252	19	137.960
Nakagawa	Natural	Landslide	44.783	142.254	24.6	311.063
Nakagawa	Natural	Landslide	44.796	142.249	12	170.068
Uryu	Planted	Undisturbed	44.367	142.248	N/A	131.310
Uryu	Natural	Undisturbed	44.391	142.203	N/A	262.313
Uryu	Planted	Landslide	44.367	142.246	22	156.737
Uryu	Natural	Landslide	44.382	142.194	11	236.254
Uryu	Natural	Landslide	44.390	142.204	21	261.495
Uryu	Natural	Landslide	44.392	142.204	21	308.410

**FIGURE 1:**
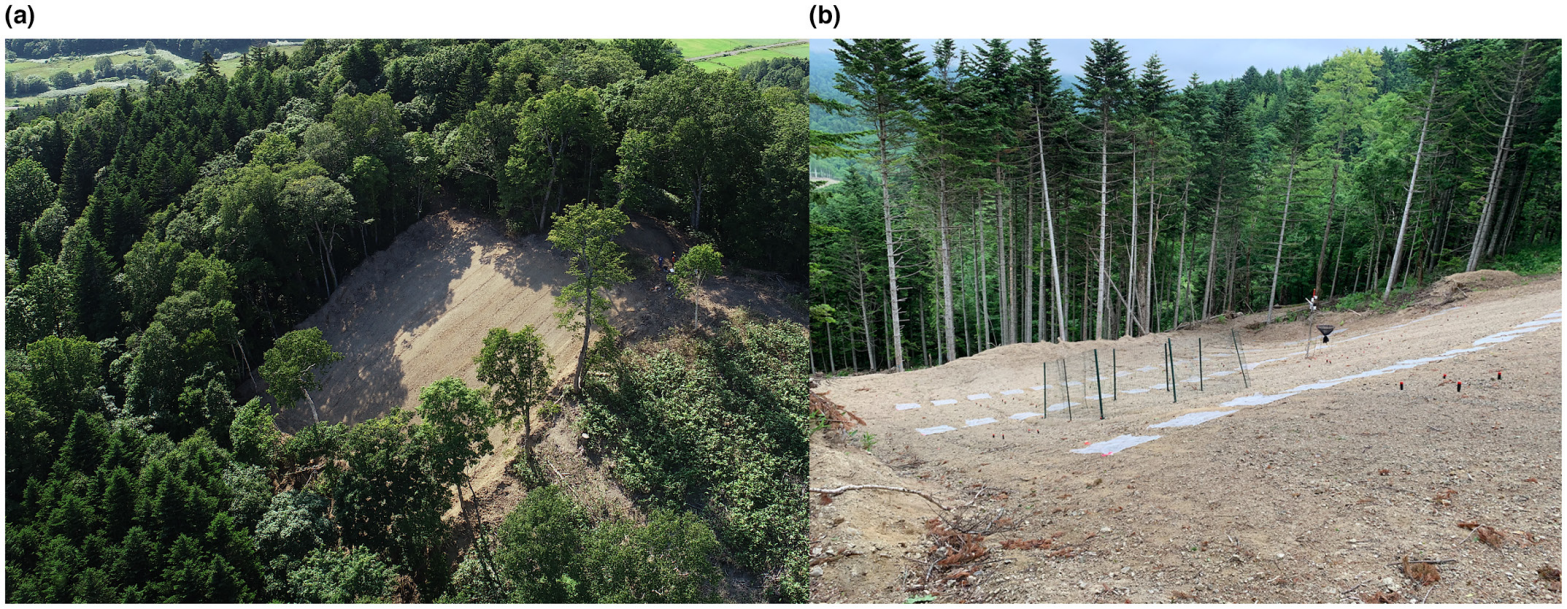
Experimental landslide sites: (a) Aerial view of a site in a natural forest; (b) view of a site in a planted forest.

Tree composition was assessed prior to experimental landslide treatment. In natural forest sites, the average basal area proportions were as follows: 19% birch, 18% oak, 18% fir, 14% linden, 12% maple, and 19% others. The forest floors were covered with dwarf bamboo, in which the coverage was more than 90%. Structures of all natural forests for the landslide and undisturbed sites were similar across localities. In all planted forest sites, more than 90% of trees were the fir *A. sachalinensis*. Their forest floors were also dominated by dwarf bamboo, but the cover was around 10%. At each landslide treatment site, all trees were cut and removed from a 35 m × 35 m area which was equally divided into four plots between December 2019 and March 2020, and in summer 2020, understory vegetation, roots, and surface soil were almost completely removed with bulldozers (Figure 1). Slope gradients after the landslide treatment ranged from 11° to 26° (20.4 ± 4.5°, mean ± SD, Table 1). In nature, landslides simultaneously create both erosional and depositional zones (Cruden & Varnes 1996; Geertsema & Pojar, 2007). This experimental treatment primarily mimicked the erosional zone of a landslide, which could be one of the most different characteristics from other disturbances. Additionally, together with the surface soil, above and belowground part of the understory vegetation, as well as the roots of overstory trees, were deposited in the area beneath each site, resembling a natural landslide depositional zone, although logs and trunks were removed from the experimental sites. This study primarily focused on ground‐dwelling beetle communities within the erosional zone that was artificially created. At each undisturbed site, a total of approximately 100 m^2^ was equally divided into four plots with at least 3 m distance separating each plot in natural and planted forests. All undisturbed sites were approximately 100 m away from the nearest treatment site and had not been disturbed artificially in at least the past 50 years.

### 2.2 Community census of ground‐dwelling beetles

We investigated ground‐dwelling beetles 1 year after the landslide treatment. For each plot (i.e., four plots in one landslide or undisturbed site), we created a two‐by‐three grid that equally divided a plot. We selected five intersections out of six intersections of the grid (i.e., approximately 4.5–5.5 m spacing between traps) and assigned each of five pitfall traps (69 mm in diameter 97 mm deep) to each of the selected intersections. Ground‐dwelling beetles were collected in late July and again in late August 2021. The first time, we collected beetles one night after trap placement, and the second time, we collected them two nights after trap placement.

A total of 587 Coleoptera individuals (excluding Staphylinidae because of the low frequency and poor condition of the samples) were identified to the species level by their morphological characteristics (Ministry of the Environment of Japan, 2022; Morimoto et al., 2007; http://www.2018jwrc‐30‐pro175.com/test/index.html), and species and abundance data were recorded (Table 2).

**TABLE 2:** Species and number of individual ground‐dwelling beetles collected in this study.

Family	Subfamily	Species	Habitat type				Total
			NAT_U	PLA_U	NAT_L	PLA_L	
Carabidae	Pterostichinae	*Synuchus melantho*	87	33	28	5	153
Carabidae	Carabinae	*Carabus opaculus*	80	17	20	0	117
Geotrupidae		*Phelotrupes laevistriatus*	36	17	3	0	56
Carabidae	Pterostichinae	*Pterostichus thunbergi*	23	29	2	0	54
Carabidae	Cicindelinae	*Cicindela sachalinensis*	0	0	37	12	49
Elateridae		*Yezohypnoidus aeneoniger*	0	0	13	11	24
Silphidae		*Silpha perforata*	20	3	0	0	23
Carabidae	Pterostichinae	*Synuchus cycloderus*	4	5	6	0	15
Carabidae	Carabinae	*Carabus exilis pararboreus*	8	6	0	0	14
Carabidae	Carabinae	*Cychrus morawitzi*	8	3	1	0	12
Carabidae	Carabinae	*Carabus gehinii*	1	8	0	0	9
Carabidae	Pterostichinae	*Pterostichus orientalis*	8	0	0	0	8
Carabidae	Pterostichinae	*Agonum chalcomum*	0	0	4	2	6
Carabidae	Callistinae	*Chlaenius variicornis*	0	1	5	0	6
Carabidae	Pterostichinae	*Pterostichus subovatus*	6	0	0	0	6
Carabidae	Carabinae	*Carabus conciliator*	0	4	0	0	4
Scarabaeidae		*Onthophagus ater*	0	4	0	0	4
Carabidae	Pterostichinae	*Colpodes buchanani*	0	0	3	0	3
Silphidae		*Nicrophorus quadripunctatus*	3	0	0	0	3
Carabidae	Bembidiinae	*Bembidion paediscum*	0	0	1	1	2
Carabidae	Broscinae	*Eobroscus lutshniki*	0	0	1	1	2
Silphidae		*Nicrophorus investigator*	0	2	0	0	2
Carabidae	Bembidiinae	*Bembidion niloticum*	0	0	0	1	1
Carabidae	Carabinae	*Carabus blaptoides rugipennis*	1	0	0	0	1
Elateridae		*Ectinus sericeus*	1	0	0	0	1
Carabidae	Loricerinae	*Loricera pilicornis*	0	1	0	0	1
Silphidae		*Phosphuga atrata*	1	0	0	0	1
Carabidae	Pterostichinae	*Pterostichus fortipes*	0	0	1	0	1
Chrysomelidae		*Sphaeroderma tarsatum*	0	0	1	0	1
Carabidae	Harpalinae	*Synuchus crocatus*	1	0	0	0	1
Carabidae	Pterostichinae	*Synuchus nitidus*	0	1	0	0	1
Unidentified			1	2	3	0	6

Abbreviations: NAT_L, landslide treatment in natural forest (*n* = 9); NAT_U, undisturbed natural forest (*n* = 3); PLA_L, landslide treatment in planted forest (*n* = 3); PLA_U, undisturbed planted forest (*n* = 3).

In addition to species composition and species diversity, we also examined body size of beetles in landslide and undisturbed sites as one of community properties. Digital images of all individuals of the collected beetles were taken with a digital camera (Tough TG‐6; Olympus). Then, the lengths of the head, pronotum, and elytron were measured, using ImageJ 1.53k. The sum of these lengths was used as body size.

### 2.3 Statistical analyses

We used Hill numbers (*q* = 0, 1, 2) to construct individual‐based rarefaction and extrapolation curves with 95% confidence intervals to compare species diversity of undisturbed communities between planted and natural forests. To examine how forest type affected community structure, we calculated the Bray–Curtis dissimilarity for species and abundance data at each undisturbed site or landslide site. In the dissimilarity calculations, 0.01 was added to all abundance data to address samples with zero abundance. Then, using the dissimilarity results, we performed a principal coordinates analysis (PCoA) to visualize differences in community structure between planted and natural forests. The analysis of multivariate homogeneity of group dispersions with 9999 permutations was performed to compare the magnitude of among‐site variability of undisturbed communities between planted and natural forests. To examine the effects of locality, forest type, and month on community composition, permutational MANOVA with 9999 permutations was performed based on the dissimilarity.

We also used Hill numbers (*q* = 0, 1, 2) to construct individual‐based rarefaction and extrapolation curves with 95% confidence intervals to compare species diversity between landslide and undisturbed communities. We calculated the Bray–Curtis dissimilarity for species and abundance data at all sites and compared the results between landslide and undisturbed communities. In the dissimilarity calculations, 0.01 was added to all abundance data to address samples with zero abundance. We performed a PCoA based on the dissimilarity to visualize differences in community structure. The analysis of multivariate homogeneity of group dispersions based on the dissimilarity with 9999 permutations was performed to compare the magnitude of among‐site variability between landslide and undisturbed communities. Permutational MANOVA with 9999 permutations was performed based on the dissimilarity to examine the effects of disturbance treatment on community structure. Among‐site variability in composition can be partitioned into spatial species turnover and nestedness of assemblages, which result from species replacement and species loss from site to site, respectively (Baselga, 2010). We partitioned among‐site variability in landslide community composition into species turnover and nestedness. In those comparisons of species diversity, composition, and variability between landslide and undisturbed communities, we combined the dataset of both planted and natural forest sites.

Body size data were fitted using a liner mixed model with treatment as a fixed effect and site as a random effect. The dataset of both planted and natural forest sites was merged. The significant test was performed, using a *F*‐test with Kenward–Roger approximation.

For all statistical analyses, we used R statistical software version 4.0.5 (R Core Team, 2021) packages “iNENT” version 2.1‐7 (Chao et al., 2014; Hsieh et al., 2016), “vegan” version 2.5‐7 (Oksanen et al., 2020), “betapart” version 1.5.4. (Baselga et al., 2021), lme4 (Bates et al., 2015), and lmerTest (Kuznetsova et al., 2017).

## 3 RESULTS

### 3.1 Species composition and abundance of the collected ground‐dwelling beetles

In total, we collected 587 (including six unidentified) individuals belonging to 31 species of ground‐dwelling beetles across all sites (Table 2). We collected a total of 422 individuals belonging to 22 species from undisturbed communities, and a total of 159 individuals belonging to 16 species from landslide communities (Table 2). Some species were only found in undisturbed sites or landslide treatment sites. For example, *Silpha perforata* and *Carabus exilis pararboreus* were repeatedly collected in undisturbed sites, whereas *Cicindela sachalinensis* and *Yezohypnoidus aeneoniger* were repeatedly collected in landslide treatment sites.

### 3.2 Effects of forest type on undisturbed and landslide communities

**FIGURE 2:**
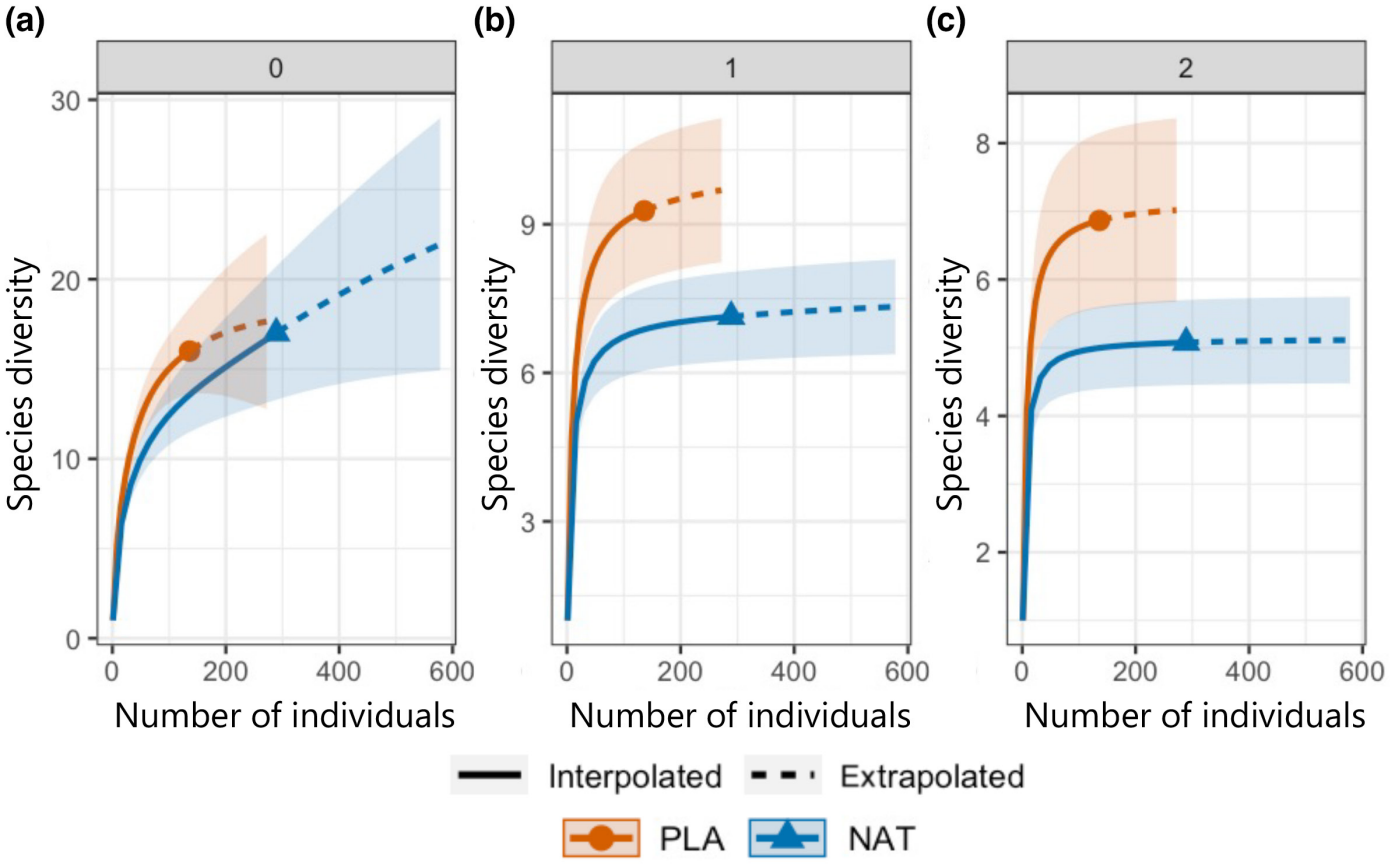
Individual‐based rarefaction curves (solid lines) and extrapolation curves (dashed lines) of species diversity in undisturbed communities in natural (NAT) and planted (PLA) forests based on three orders of Hill numbers: (a) *q* = 0 (species richness), (b) *q* = 1 (Shannon diversity), and (c) *q* = 2 (Simpson diversity).

The individual‐based rarefaction and extrapolation curves indicated no significant differences in species diversity of undisturbed communities between planted and natural forests for *q* = 0; thus, they did not differ in species richness. However, for *q* = 1 (Shannon diversity) or 2 (Simpson diversity), species diversity was greater in planted forests (Figure 2).

**FIGURE 3:**
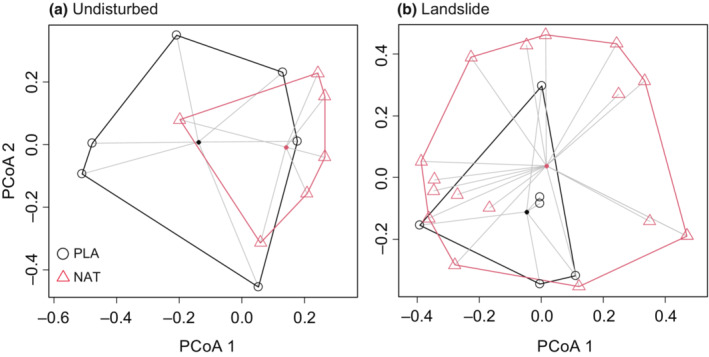
Principal coordinate analysis (PCoA) plots of the compositions of ground‐dwelling beetle communities for the two forest types based on the Bray–Curtis dissimilarity: (a) Undisturbed community and (b) landslide community. Unfilled circles and unfilled triangles represent communities in planted forests and in natural forests, respectively. Filled circles represent the spatial centroids of each group.

The magnitude of among‐site variability in species composition was similar between undisturbed planted and natural forests (Figure 3a; Permutation test, *F* = 0.08, *p* = .76).

Effects of locality, forest type, and month on community composition were significant at undisturbed sites (Table 3). In contrast, no significant effect of forest type (i.e., pre‐disturbance vegetation) on landslide communities was detected (Table 3, Figure 3b). Thus, pre‐disturbance vegetation did not influence the community structure of ground‐dwelling beetles after a landslide disturbance.

**TABLE 3:** Effects of locality, forest type, and month on the ground‐dwelling beetle community.

Undisturbed/landslide	Variables	*F*	*p*
Undisturbed sites	Locality (L)	5.00	**.0012**
	Forest type (Ft)	5.80	**.0005**
	Month (M)	8.42	**.0002**
	L × Ft	3.02	**.0148**
	Ft × M	3.35	**.0185**
	M × L	3.90	**.0043**
Landslide sites	Locality (L)	3.52	**.0004**
	Forest type (Ft)	1.84	.0830
	Month (M)	5.40	**.0001**
	L × Ft	1.20	.2814
	Ft × M	0.74	.6449
	M × L	2.30	**.0107**

*Note*: Significant values are indicated in bold.

### 3.3 Comparison of structure and species diversity between undisturbed and landslide communities

**FIGURE 4:**
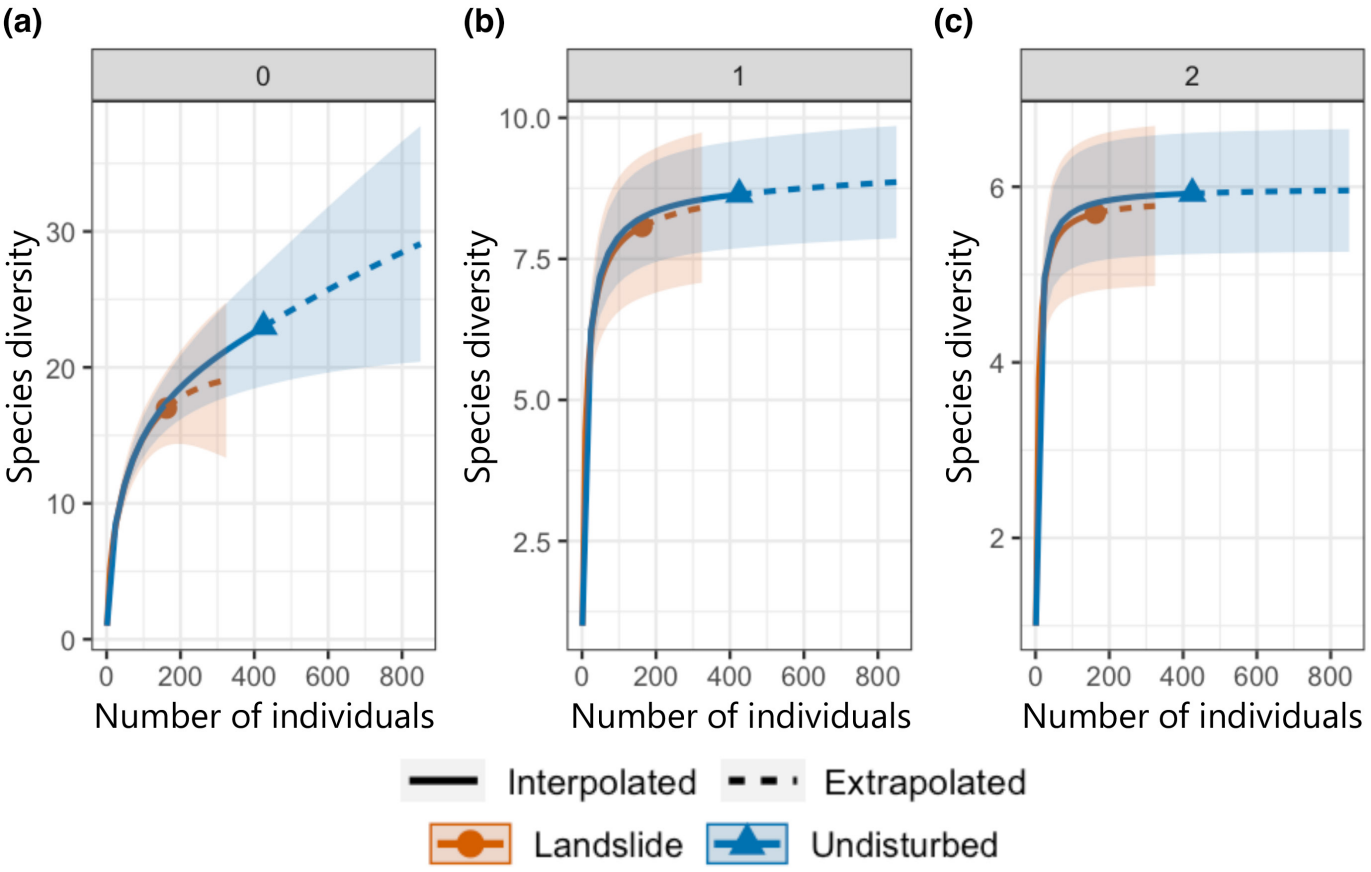
Individual‐based rarefaction curves (solid lines) and extrapolation curves (dashed lines) of species diversity in landslide and undisturbed communities based on three orders of Hill numbers: (a) *q* = 0 (species richness), (b) *q* = 1 (Shannon diversity), and (c) *q* = 2 (Simpson diversity).

The individual‐based rarefaction and extrapolation curves indicated no significant differences in species diversity between undisturbed and landslide communities (Figure 4).

**FIGURE 5:**
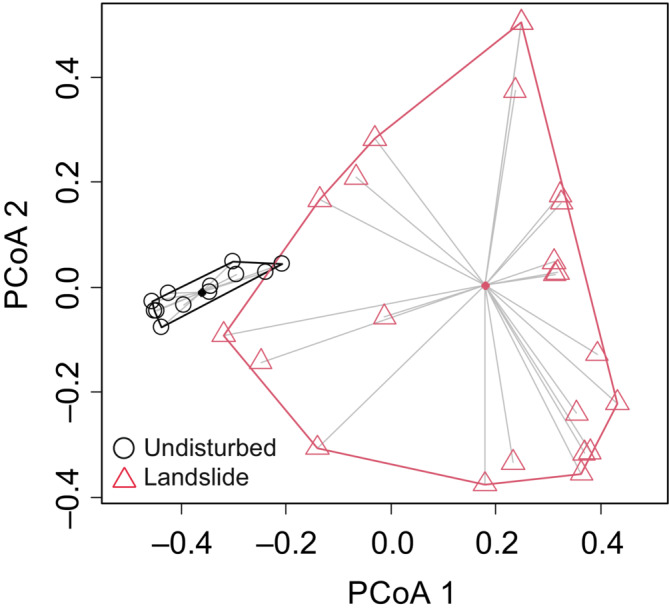
A principal coordinate analysis (PCoA) plot of the compositions of ground‐dwelling beetle communities based on the Bray–Curtis dissimilarity. Unfilled circles and unfilled triangles represent undisturbed communities and landslide communities, respectively. Filled circles represent the spatial centroids of each group.

However, landslide treatment drastically changed the community structure (Figure 5; PERMANOVA, *F* = 10.69, *p* = .0001). The PCoA results illustrated that not only community composition but also among‐site variability clearly differed between landslide and undisturbed sites. Among‐site variability was much greater at landslide sites than at undisturbed sites (Figure 5; Permutation test, *F* = 14.67, *p* = .001). The average distance from the centroid of each landslide and undisturbed group in the PCoA result was 4.1 times greater in landslide communities. Community turnover analysis showed that the high among‐site variability of landslide sites was caused primarily by species turnover (0.82) rather than by nestedness (0.09).

**FIGURE 6:**
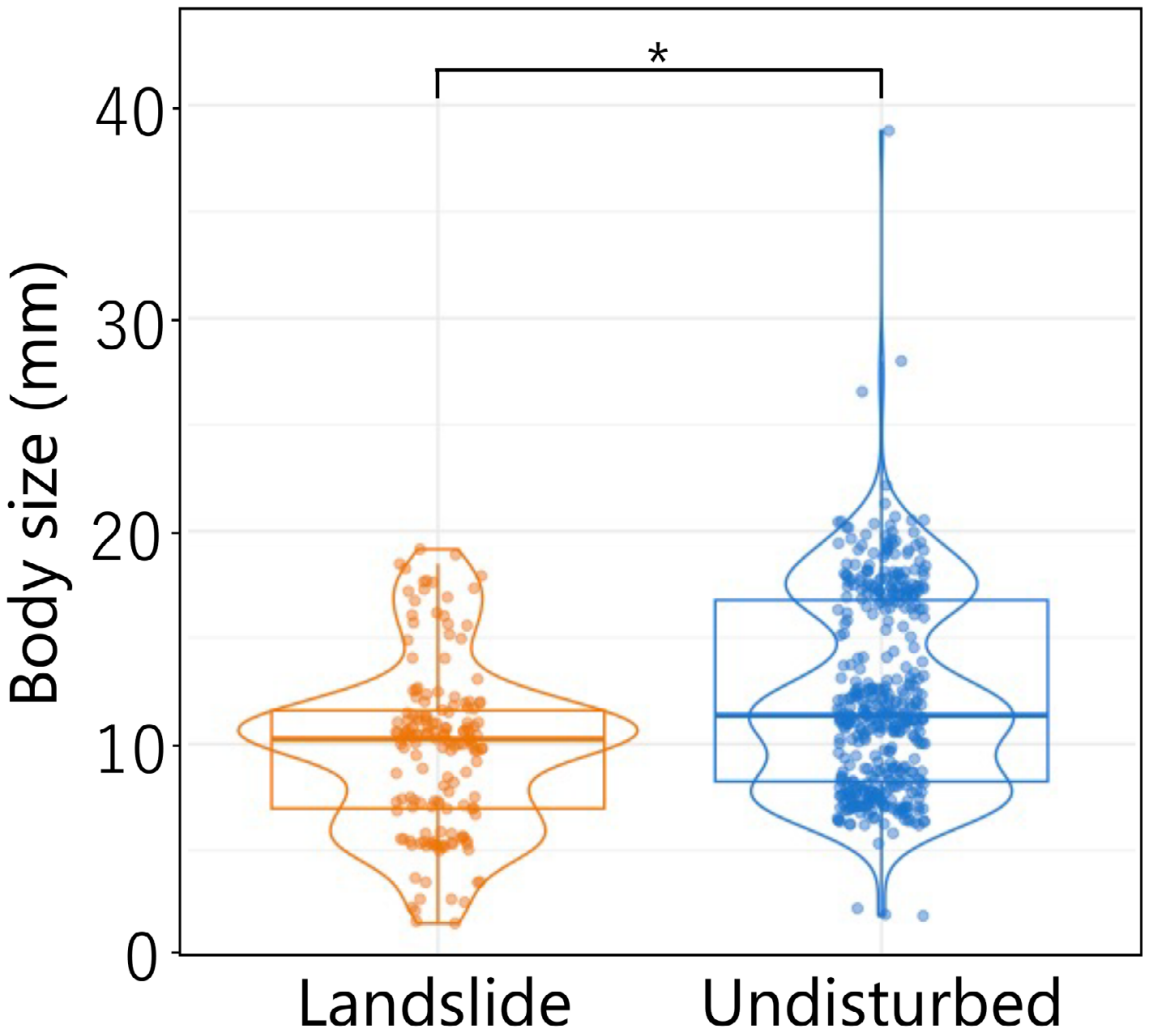
Body size of ground‐dwelling beetles in landslide and undisturbed sites. Each point indicates body size of each individual. An asterisk represents significant difference between landslide and undisturbed sites (*p* < .05).

The body size of ground‐dwelling beetles collected in landslide sites was significantly smaller than in undisturbed sites (Figure 6; *F*‐test, *p* = .015). When beetle species identity as a random effect was added to the model, effect of landslide treatment on body size was not significant (*p* = .79). This indicates the difference in body size was due to community composition rather than intraspecific variation.

## 4 DISCUSSION

This study clearly illustrated community assembly of ground‐dwelling beetles in response to landslide disturbance. Although pre‐disturbance vegetation did not affect the community structure after landslides, species composition greatly differed between landslide and undisturbed communities. These results indicate that a niche‐selection process shaped the ground‐dwelling beetle communities that colonized sites after a landslide. Furthermore, among‐site variability in species composition (i.e., *β*‐diversity) was much greater at landslide sites, whereas species diversity was comparable between landslide and undisturbed sites. Overall, this study suggests that a stochastic process of ecological drift, as well as niche selection, is critical for community assembly after a landslide.

### 4.1 The structure of undisturbed ground‐dwelling beetle communities

To clarify the structure of the communities disturbed by landslides, the fundamental structure of the undisturbed communities must be understood as a comparison. Therefore, we first describe the characteristics of the undisturbed forest communities.

The structure of undisturbed forest communities, but not that of landslide‐disturbed communities, differed by forest type (planted or natural; Table 3). The species richness of undisturbed ground‐dwelling beetle communities was comparable between planted and natural forests (Figure 2a), but the evenness was greater in planted forests (Figure 2a,b). This greater evenness in planted forests is likely because planted forests are a more stable and homogeneous environment than natural forests. Greater environmental variability in terms of temperature, intensity of ambient insolation, and wind speed may decrease evenness in arthropod communities (Larrivée & Buddle, 2009). Previous studies have reported that carabid beetle communities have lower species richness in conifer plantations than in natural deciduous broadleaf forests regardless of the age of plantations (5–50 years) (Magura et al., 2003; Yu et al., 2006). On the other hand, comparative studies among conifer plantations and semi‐natural or secondary deciduous broadleaf forests have showed mixed results. For example, Fahy and Gormally (1998) and Kaizuka et al. (2020) have reported that carabid beetle communities have lower species richness in conifer plantations than in semi‐natural and secondary deciduous broadleaf forests, whereas Fuller et al. (2008) and Zou et al. (2019) reported no difference in species richness between forest types. For the results of our study, there are two explanations. First, our natural forest sites were not old‐growth forests. This may explain the reason why species richness exhibited no difference between planted and/or natural forests, as found by some previous studies that compared conifer plantations with semi‐natural and secondary deciduous broadleaf forests. Second, our planted forest sites were not large and surrounded by natural mixed forest. Thus, the local ground‐dwelling beetle communities in planted and natural forests are likely part of a metacommunity in a continuous mixed‐forest landscape with both coniferous and broadleaf trees. Although our planted forest sites included only a single species of conifer (*A. sachalinensis*), we found beetle species known to be broadleaf forest specialists, *Carabus opaculus* (Kaizuka et al., 2020), *Phelotrupes laevistriatus*, and *Carabus gehinii* (Kaizuka et al., 2020), at undisturbed planted forest sites (Table 2). In addition, conifers (*A. sachalinensis*) often occur in the natural forest. Thus, beetle species may frequently be dispersed across natural and planted forest patches in a continuous, mixed‐forest landscape, and this dispersal would maintain comparable levels of species diversity in the beetle communities between planted and natural forest sites. These explanations were not mutually exclusive. The similar among‐site variability in beetle species composition between the planted and natural forest sites may also support the existence of a metacommunity where species dispersal occurs across both the uniform environment of planted forest patches and heterogeneous natural forest environments. In addition, the differences in community composition between the planted and natural forest sites were not large (as shown by the considerable overlap in Figure 3a).

### 4.2 Effect of pre‐disturbance vegetation on the landslide communities

To our knowledge, this is the first study to demonstrate experimentally that pre‐disturbance vegetation had no effect on the structure of communities disturbed by landslides (Table 3, Figure 3b). Iida et al. (2021) showed an effect of pre‐disturbance vegetation on the arthropod community structure after a volcanic eruption; they suggested that the presence or absence of post‐disturbance litter deposition, which varies with the pre‐disturbance vegetation, affects the community structure. In contrast, we observed very little litter deposition at our landslide sites because only 1 year had passed since the complete removal of the surface soil. This lack of litter deposition may explain why the pre‐disturbance vegetation had no effect on the structure of the ground‐dwelling beetle communities in our data. However, other taxa, in particular plant taxa, may be affected by the pre‐disturbance vegetation during the community assembly process because of seed and propagule dispersal from the surrounding vegetation (i.e., pre‐disturbance vegetation). Therefore, it is likely that during the vegetation recovery stage, the ground‐dwelling beetle community would also be indirectly affected by the pre‐disturbance vegetation via the recovered vegetation.

No differences in landslide communities between pre‐vegetation types would result from immigration processes in assembly. Most of species were only collected from either landslide or undisturbed sites, whereas a few common species were collected from both sites (Table 2). This suggests two processes in immigration of ground‐dwelling beetles to landslide sites: (1) common species immigrating from neighboring forests, (2) species immigrating from habitats that are similar to our landslide sites. For the former, a few dominant species, which inhabit both planted and natural forest areas, colonized the landslide sites (Table 2). For the latter, many species with a high dispersal capacity, which are likely to prefer the landslide‐like habitats such as non‐forested gravel environments (e.g., the tiger beetle *C*. *sachalinensis* and the click beetle *Y*. *aeneoniger*), colonized the landslides sites. However, the majority of species were from the latter group. This is likely to be because landslides created a harsh environment characterized by higher temperatures, lower humidity, and stronger sunlight compared with the neighboring forests. Therefore, niche selection probably plays a critical role in community assembly at landslide sites, as discussed below.

### 4.3 Niche selection and stochasticity in response to landslides

Previous studies have reported that species richness of ground‐dwelling beetles increases in communities immediately after disturbances such as windthrows (Bouget, 2005), forest fires (Paquin, 2008), and clear‐cutting (Heliölä et al., 2001; Koivula et al., 2002; Yu et al., 2006). Our experimental study yielded similar results in that the overall species diversity of ground‐dwelling beetles was not decreased in the early recovery stage (i.e., 1 year after landslides) when landslide communities were compared with undisturbed communities.

However, the fact that the species composition of the communities differed significantly between post‐landslide and undisturbed sites suggests that ecological filtering from the regional species pool likely differed between the landslide and undisturbed forest sites. The absence of a forest‐type effect on landslide communities, despite the existence of such an effect on the undisturbed community structure, supports this interpretation. Therefore, niche selection has a critical role in shaping landslide communities of ground‐dwelling beetles. In the mixed forests of northern Hokkaido, there are many sites with habitats similar to our landslide sites, including natural small‐landslide sites, gravel river banks, and sites that have been artificially disturbed by logging and soil scarification (Aoyama et al., 2011). Because biotic and abiotic environments in these habitats may be similar to those of landslide sites (e.g., no vegetation, bare surface soil, or gravel), beetle populations in these habitats are likely to be sources of newly colonizing beetles, such as *C. sachalinensis*, *Bembidion paediscum*. *Cicindela sachalinensis*, and *B. paediscum*, which are known to prefer forest road, bare surface soil, and gravel river bank habitats (Morimoto et al., 2007; Ueno et al., 1985). Patchy environments similar to our landslide habitats exist widely within a natural forest, and dispersal from the metacommunity around these landscapes is not restricted due to natural forest matrix. Therefore, beetle species from the metacommunity can immediately colonize landslide sites and maintain species diversity, with niche selection having a strong effect due to the harsh environmental conditions.

On the other hand, the among‐site variability was 4.1 times higher for landslide sites than for undisturbed sites because of high species turnover. In fact, many of the species collected at the landslide treatment sites were collected from only a few sites, and no species were found across all landslide sites. The high site‐to‐site variability and high turnover suggest that stochastic colonization by species filtered by the harsh post‐landslide environment predominantly affected community structure at the landslide sites. Previous studies have reported an increase in the number of species with small body size and high dispersal capacity in arthropod communities after a disturbance (Bailey et al., 2018; Butterfield et al., 1995; Rainio & Niemelä, 2003). In our experiment, the body size of the beetle community was also smaller at landslide sites than at undisturbed sites (Figure 6), suggesting colonization by beetle species with a high dispersal capacity. Thus, beetle species with a high dispersal capacity inhabiting similar types of habitats (e.g., gravel river banks, logging sites, and forest roads) are likely to immigrate to the scattered landslide sites in a stochastic manner. Alternatively, the different environmental filter due to the difference in the environment at each landslide site may explain the high site‐to‐site variability and high species turnover. However, the site‐to‐site variability in undisturbed communities was much lower than landslide sites while environmental heterogeneity among undisturbed sites may be greater than landslide sites. Thus, differences in environmental conditions among landslide sites would unlikely explain the large community variability. Overall, these results suggest that the landslide treatment not only caused deterministic processes but also strengthen stochastic processes in community assembly in comparison with undisturbed sites, at least in the early post‐landslide stage.

Previous studies have shown that patch connectivity and habitat heterogeneity are important for the maintenance of ground‐dwelling beetle populations (Duflot et al., 2014; Neumann et al., 2016; Niemelä, 2001). Our results also likely reflect both the presence of environments such as river banks and logging sites around our study sites (i.e., habitat heterogeneity) and connectivity between such sites and our experimental sites. In other words, the surrounding landscape structure, including the degree of patch connectivity, the distance between patches, and the diversity of patch environments (i.e., the number of different insect habitat types) may strongly influence the recovery rate of species diversity after a disturbance.

This study yielded new insights into community assembly by comparing communities between landslide and undisturbed treatments at replicate sites in a large‐scale field experiment. However, the results show only the immediate responses of the beetle communities. In the future, further temporal changes in the communities at these sites need to be investigated. In addition, the dispersal abilities of the species that colonize the landslide sites should be investigated. Further, the contributions of patch connectivity, patch diversity, and patch distribution to the recovery of species diversity after a landslide should be quantified. For achieving these aims, large‐scale manipulative field experiments with replicate sites have much to offer.

## AUTHOR CONTRIBUTIONS

**Jumpei Furusawa:** Conceptualization (equal); data curation (lead); formal analysis (equal); investigation (equal); methodology (equal); validation (lead); visualization (equal); writing – original draft (equal); writing – review and editing (equal). **Kobayashi Makoto:** Conceptualization (equal); investigation (supporting); methodology (equal); writing – review and editing (supporting). **Shunsuke Utsumi:** Conceptualization (lead); data curation (supporting); formal analysis (supporting); funding acquisition (lead); investigation (supporting); methodology (equal); project administration (lead); supervision (lead); validation (supporting); visualization (supporting); writing – review and editing (lead).

## CONFLICT OF INTEREST STATEMENT

The authors declare that they have no conflict of interest.

### OPEN RESEARCH BADGES

This article has earned an Open Data badge for making publicly available the digitally‐shareable data necessary to reproduce the reported results. The data is available at https://doi.org/10.6084/m9.figshare.22210723.

## Data Availability

The data that support the findings of this study are openly available in figshare at https://doi.org/10.6084/m9.figshare.22210723.
