# CORRIGENDUM

**DOI:** 10.1002/ece3.10385

**Published:** 2023-07-26

In the recent article by Furusawa et al. (2023), some data points in Figure 6 were incorrectly represented due to typographical errors. Corrected Figure 6 and related statistic values are as follows:

**FIGURE 6:**
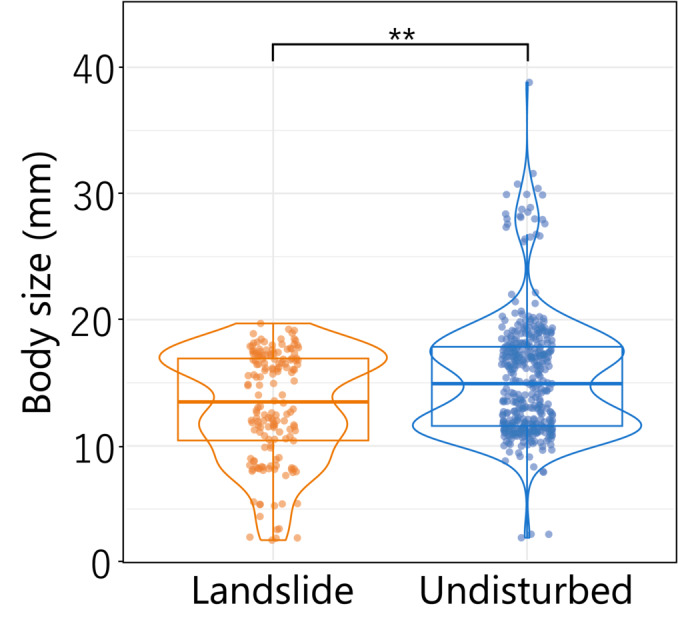
Body size of ground‐dwelling beetles in landslide and undisturbed sites. Each point indicates body size of each individual. Asterisks represent significant difference between landslide and undisturbed sites (*p* < .01).

The first and second sentences in the third paragraph of the result section 3.3 should be replaced by “The body size of ground‐dwelling beetles collected in landslide sites was significantly smaller than in undisturbed sites (Figure 6; *F*‐test, *p* = .007). When beetle species identity as a random effect was added to the model, effect of landslide treatment on body size was not significant (*p* = .87).”

We apologize for the errors. Other parts of the article, including any interpretations, discussions, and conclusions, are not affected by these corrections.
